# First-line chemotherapy with docetaxel plus capecitabine followed by capecitabine or hormone maintenance therapy for the treatment of metastatic breast cancer patients

**DOI:** 10.3892/ol.2014.2787

**Published:** 2014-12-10

**Authors:** XU LIANG, YING YAN, LINA WANG, GUOHONG SONG, LIJUN DI, HANFANG JIANG, CHAOYING WANG, HUIPING LI

**Affiliations:** Key Laboratory of Carcinogenesis and Translational Research (Ministry of Education), Department of Breast Oncology, Peking University Cancer Hospital and Institute, Beijing 100142, P.R. China

**Keywords:** capecitabine, maintenance therapy, docetaxel, metastatic breast cancer

## Abstract

The primary aim of the present study was to evaluate whether maintenance therapy with capecitabine or hormone replacement therapy (HRT) results in improved progression-free survival (PFS) in metastatic breast cancer (MBC) patients who had previously achieved disease control with first-line docetaxel plus capecitabine (TX) chemotherapy. Seventy-nine metastatic breast cancer patients treated between January 2008 and June 2013 with TX chemotherapy were retrospectively analyzed. Following successful initial disease control by the combination chemotherapy, 39 patients received single-agent capecitabine maintenance therapy and 40 patients received HRT as maintenance therapy. The PFS time, objective response rate, clinical benefit rate and safety of the two groups were compared. The median PFS of the total cohort (n=79) was 11.0 months. Furthermore, the median PFS time of the capecitabine (n=39) and HRT groups (n=40) were 10.9 and 11.1 months, respectively (P=0.283). Compared with the PFS time of maintenance treatment only, single-agent capecitabine treatment following TX chemotherapy prolonged the PFS time by 6.8 months and HRT following TX chemotherapy prolonged PFS time by 5.8 months (P=0.551). Of the total cohort, 49 patients did not receive palliative endocrine therapy prior to chemotherapy, including 22 patients in the capecitabine maintenance group and 27 patients in the HRT maintenance group. The PFS time from the commencement of maintenance treatment was significantly different between the two groups, 6.1 months in the capecitabine group compared with 11.5 months in the HRT group (P=0.045). For the 30 patients who underwent palliative endocrine therapy prior to TX chemotherapy, the PFS times of the capecitabine and HRT maintenance treatment groups were 7.5 and 4.1 months, respectively (P=0.043). However, the occurrence of adverse events, such as hematological and gastrointestinal toxicity, as well as hand-foot syndrome, were not significantly different between the two groups. The current study indicated that single-agent capecitabine maintenance therapy may be a potential treatment strategy for MBC patients who responded to capecitabine-based chemotherapy. In particular, capecitabine may provide a more effective maintenance treatment duration compared with HRT for patients who had previously undergone first-line palliative HRT for MBC.

## Introduction

Breast cancer represents the most common type of malignancy in females, worldwide. Despite earlier diagnosis and improvement in adjuvant therapies, a number of patients present with metastatic recurrence, which has a two to three year median overall survival time (1,2). Hormonal therapy, chemotherapy and more recently biological treatment are systemic therapies designed to reduce the size of tumors, improve patient survival and preserve quality of life. However, in a metastatic setting, the majority of patients will relapse regardless of the initial efficacy of the treatment strategy undertaken. The most important therapeutic goals in metastatic breast cancer (MBC) are palliative and aim to improve progression free survival (PFS). However, this management of MBC is a clinical challenge for healthcare workers, as the optimal type and duration of chemotherapy, and the benefits of maintenance chemotherapy versus maintenance hormonal treatment required, have yet to be determined. Thus, the present retrospective study aimed to investigate the impact of HRT and capecitabin, two types of maintenance therapy, on MBC patient PFS.

Following a response to rescue chemotherapy, maintenance treatment with HRT or targeted agents may be considered for the treatment of MBC; however, maintenance HRT is limited to MBC patients with hormone receptor-positive disease (3,4). A number of targeted agents are widely accepted as a type of maintenance therapy for MBC, for example trastuzumab is administered for human epidermal growth factor receptor 2 (Her-2)-positive MBC (5,6). However, targeted agents are relatively high in cost and, thus, are not routinely selected as maintenance treatment in developing countries. In addition to efficacy, the convenience and tolerability of the maintenance treatment must be considered; for example, intravenous chemotherapy requires frequent hospital visits for the patient, which are associated decreased quality of life for patients and increased healthcare worker costs. Therefore, the majority of patients prefer oral as opossed to intravenous chemotherapy (7,8), for example oral capecitabine.

Capecitabine is approved by the US Food and Drug Administration for the treatment of patients with locally advanced breast cancer or MBC. It has a favorable safety profile with adverse events effectively managed by dose modification (9) and it can conveniently be administered by oral dosing (10). Furthermore, capecitabine typically lacks cumulative toxicity with prolonged use and, thus, is suitable for long-term administration. A number of clinical trials of capecitabine for the treatment of MBC indicate that capecitabine is effective when combined with a variety of agents, including taxanes, vinorelbine, gemcitabine, trastuzumab or bevacizumab (11–16). However, it is unclear how the therapeutic effects of capecitabine-based first-line combination chemotherapy may be maintained. Thus, the current study presents the results of an analysis of MBC patients receiving capecitabine or hormone replacement therapy (HRT) as maintenance treatment following initial response to capecitabine-based combination therapy.

## Patients and methods

### Patient selection

From January 2008 to June 2013, 226 MBC patients received TX combination therapy at the Department of Breast Oncology of Beijing Cancer Hospital (Beijing, China). Of these, 79 patients were eligible to receive maintenance treatment according to the following inclusion criteria: Female patients aged ≥18 years with histologically confirmed primary breast cancer; patients must have a minimum of one measurable lesion, according to Response Evaluation Criteria in Solid Tumors guidelines (RECIST) 1.0 (17), and an Eastern Cooperative Oncology Group score of ≤2 (18); patients must not have undergone prior chemotherapy for advanced disease; and patients must have completed four to eight cycles and achieved disease control [complete relief (CR), partial relief (PR) or stable disease (SD)]. Furthermore, patients were allowed to receive one-line endocrine treatment for advanced disease prior to docetaxel plus capexitabine (TX) chemotherapy. This study was approved by the ethics committee of Beijing Cancer Hospital (Beijing, China) and written informed consent was obtained from all patients.

### Treatment strategy

Capexitabine was administered as the combination and maintenance therapy at a dose of 1,000 mg/m^2^ twice daily on days 1–14 followed by a 7-day rest period. In the combination regimen, docetaxel was coadministered, as a 1-h 75 mg/m^2^ intravenous infusion on day 1 of every 3-week cycle. Following a response to chemotherapy, 39 patients continued to receive single-agent capecitabine with the abovementioned dose, whilst 40 patients received hormonal therapy with tamoxifen (n=3), toremifene (n=7), exemestane (n=15), letrozle (n=6) or anastrozole (n=9). To relieve the symptoms of hand-foot syndrome during maintenance therapy, all patients were coadministered with 100 mg vitamin B6 three times daily.

### Efficacy and safety assessments

The PFS time of the 79 patients was determined as the interval from the day of combined TX chemotherapy commencement to cancer progression, cancer-related mortality, mortality from an unknown cause during therapy, or the final day of follow-up for patients who had not progressed at the date of analysis. By contrast, for maintenance treatment PFS, the start time was defined as the day of capecitabine or hormonal agent maintenance therapy commencement. Additionally, the clinical efficacy and major adverse events were investigated, with response assessed using RECIST and adverse events graded according to the National Cancer Institute Common Toxicity Criteria version 3.0 (19).

### Statistical analysis

The duration of response was defined as the period between CR or PR onset and evidence of disease progression, and the duration of response and PFS were estimated using the Kaplan-Meier method. Additionally, the baseline characteristics of the patients and the incidence of adverse events between capecitabine and HRT maintenance therapy were compared using Pearson’s χ^2^ test. All statistical analyses were performed using SPSS software (version 15.0; SPSS Inc., Chicago, IL, USA). P<0.05 was considered to indicate a statistically significant difference.

## Results

### Patient characteristics

The 79 patients investigated in the present study were divided into two groups, with 39 patients receiving capecitabine maintenance therapy and 40 patients receiving hormone maintenance therapy. The baseline patient characteristics of the 79 patients are summarized in Table I. The median patient age was 55 years (range, 34–75 years), the majority of patients exhibited hormone receptor-positive tumors (79.8%; HR-positive status indicates estrogen receptor-positive and/or progesterone receptor-positive), and Her-2-negative disease (83.5%). The most common sites of metastasis were the bone and lung (51.9%). The majority of patients had received prior anthracycline-based chemotherapy (78.4%), with more than half (50.6%) receiving prior taxane-based chemotherapy. Additionally, palliative hormonal therapy due to metastasis had been administered prior to DX chemotherapy in 30 patients, including 17 patients (43.5%) in the capecitabine maintenance group and 13 patients (32.5%) in the HRT group. Of the 40 patients who received endocrine agent maintenance, 28 patients received aromatase inhibitors (AIs), five patients received toremifene, six received goserelin plus AIs and one patient received tamoxifene.

### Efficacy of combined DX chemotherapy plus maintenance treatment

**Figure 1:**
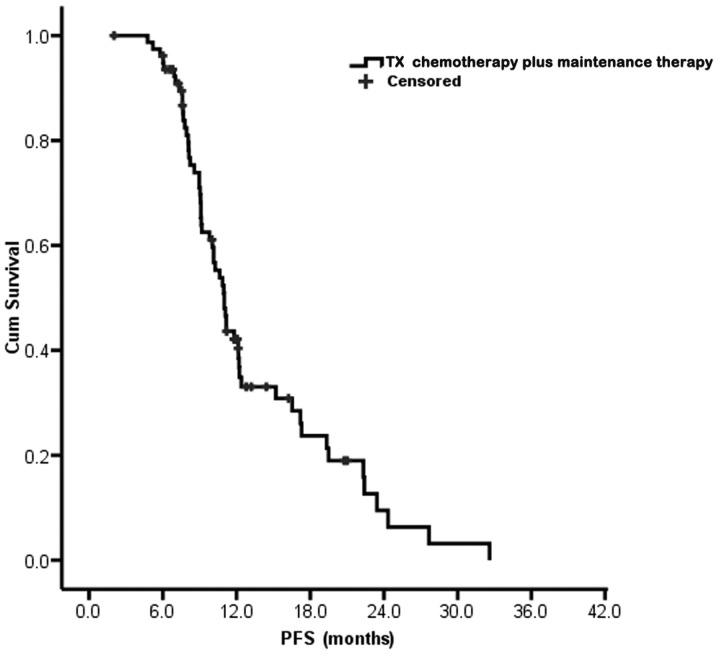
Median PFS of 79 patients who underwent combined chemotherapy followed by maintenance treatment. PFS, progression-free survival; XD, docetaxel plus capecitabine.

Combined agents chemotherapy plus maintenance therapy was received by all 79 patients and resulted in a median PFS of 11.0 months [95% confidence interval (CI), 10.1–11.9 months; Fig. 1]. Dependent on the nonprogressive response, eight patients (10.1%) received eight cycles of combined chemotherapy, 14 patients (17.7%) received four cycles and 57 patients (72.2%) recevied six cycles. The baseline response to the combination chemotherapy was a CR in two patients (2.5%), a PR in 32 patients (40.5%) and SD in 45 patients (57.0%). For the 39 patients following the single-agent capecitabine maintenance treatment, the baseline was as follows: Two patients (5.1%) achieved a CR, 20 patients (51.3%) exhibited SD and PR occured in 17 patients (43.6%), whilst in the 40 HRT patients, PR occured in 15 patients (37.5%) and SD in 25 patients (62.5%). The rate of CR and PR were not significantly different between the two groups (48.7 vs. 37.5%, respectively; P=0.314).

### Efficacy of capecitabine maintenance therapy and HRT

**Figure 2:**
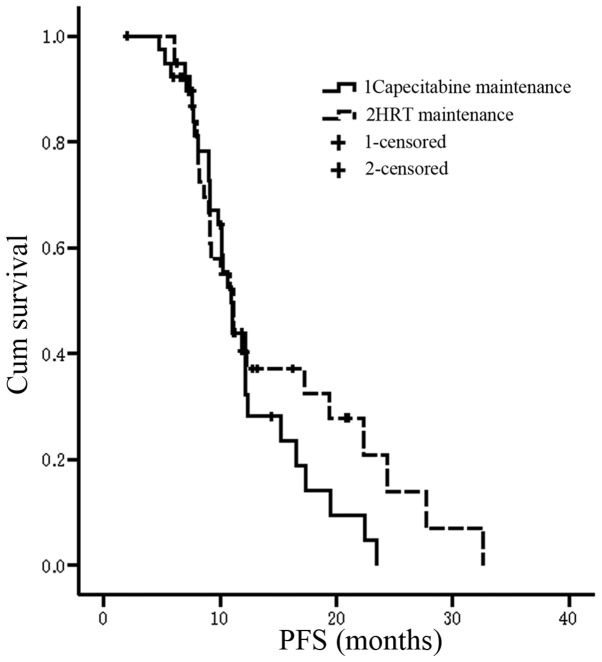
PFS of docetaxel plus capecitabine chemotherapy followed by two types of maintenance therapy. HRT, hormone replacement therapy; PFS, progression-free survival.

**Figure 3:**
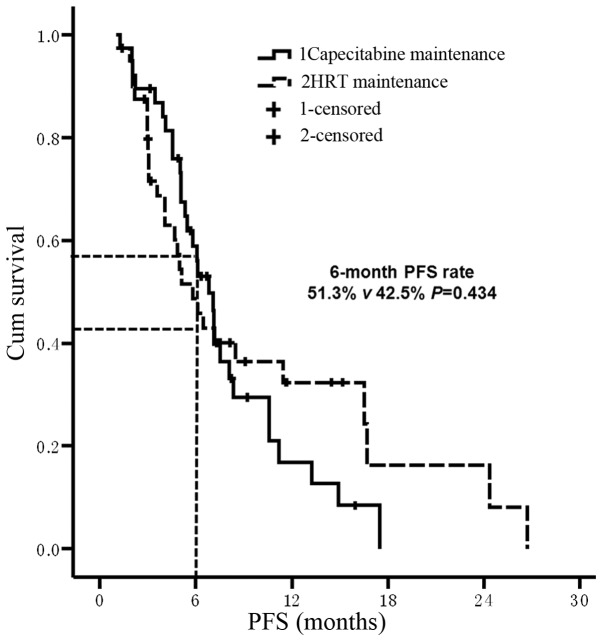
PFS of maintenance therapy in two groups. HRT, hormone replacement therapy; PFS, progression-free survival.

The median PFS time of patients in the TX chemotherapy plus capecitabine maintenance therapy group was 10.9 months (95% CI, 9.9–12.0 months) and for the TX chemotherapy plus HRT group was 11.1 months (95% CI, 8.8–13.4 months; P=0.28; Fig. 2). Compared with the PFS time of maintenance treatment only, TX chemotherapy plus single-agent capecitabine treatment prolonged survival by 6.8 months (95% CI, 5.7–7.9 months), which was not significantly different to the PFS time of TX chemotherapy plus HRT (5.8 months; 95% CI, 4.0–7.6 months; P=0.55; Fig. 3). The 6-month PFS rate of the two types of maintenance treatment were similar (95% CI, 51.3 for capecitabine vs. 42.5% for HRT; P=0.434).

### Efficacy of maintenance therapy with or without palliative endocrine therapy prior to chemotherapy

**Figure 4:**
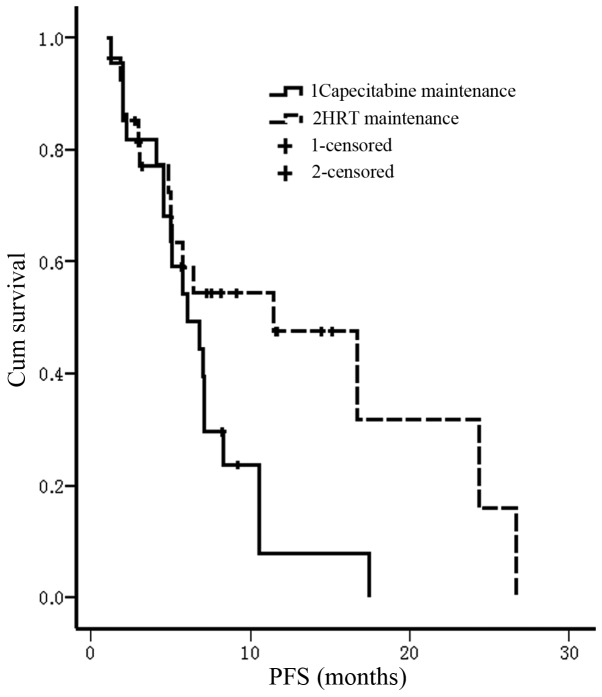
PFS of maintenance therapy for patients who did not undergo palliative hormonal therapy as the first-line therapy. HRT, hormone replacement therapy; PFS, progression-free survival.

**Figure 5:**
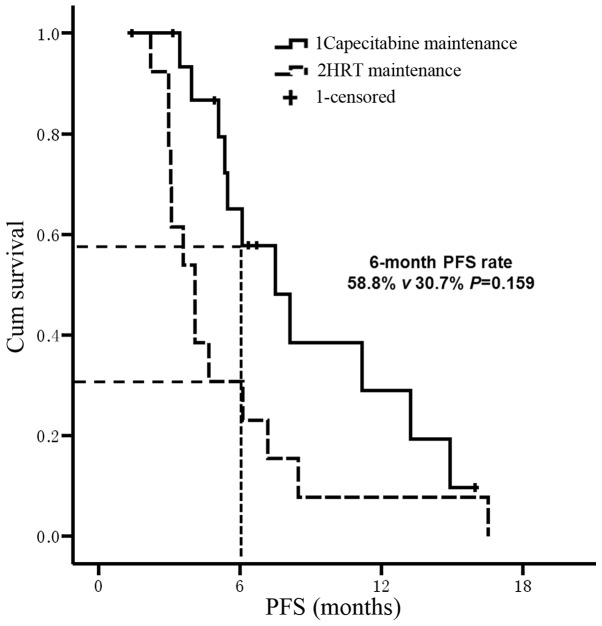
PFS of maintenance therapy for patients with palliative hormonal therapy as the first-line therapy. HRT, hormone replacement therapy; PFS, progression-free survival.

In 49 patients, the first-line treatment strategy was not palliative hormonal therapy; this included 22 patients in the capecitabine maintenance group and 27 patients in the HRT maintenance group. For these 49 patients, the median PFS time from maintenance treatment was 6.1 months in the capecitabine group and 11.5 months in the HRT group (P=0.045; Fig. 4). Prior to the administration of TX chemotherapy, 30 patients had received palliative hormonal therapy as first-line therapy for metastatic breast cancer, including 17 patients in the capecitabine maintenance group and 13 in the HRT group. The median PFS time of these 30 patients from maintenance treatment was 7.5 months in the capecitabine group and 4.1 months in the HRT group (P=0.043; Fig. 5). Furthermore, the 6-month PFS rate was 58.8% in the capecitabine maintenance group and 30.7% in the HRT group (P=0.159; Fig. 5). No significant difference was identified between the two maintenance groups; however, this may have been due to an insufficient number of cases being investigated, as it was observed that the 6-month PFS rate of the capecitabine maintenance group was almost twice that of the HRT group.

### Toxicity analysis

Table II indicates the treatment-associated toxicities [according to National Cancer Institute Common Terminology Criteria for Adverse Events (19)] of 79 patients observed in the present study. Hematologic and gastrointestinal toxicities, as well as hand-foot syndrome did not occur at significantly different rates in the two groups. For example, the rate of grade III neutropenia was marginally higher in the capecitabine maintenance group compared with the HRT group (20.5 vs. 10.0%, respectively; P=0.225), and the mean incidence of hand-foot syndrome was markedly greater in the capecitabine group compared with the HRT group (48.7 vs. 27.5%, respectively; P=0.052).

## Discussion

The long-term survival of female MBC patients remains poor, despite decades of research into systemic therapy (20). Systemic therapy uses chemotherapy or hormonal therapy, depending on factors, such as hormone receptor status, performance status, disease bulk, number of disease sites and patient age. For HR-positive patients, initial chemotherapy may be selected as the treatment modality due to the aggressive nature of the disease; in particular, combination chemotherapy has demonstrated a number of potential benefits, including an increased therapeutic response, a shorter time to progression and the possibility of improved overall survival. Thus, chemotherapy is often selected as the the priority treatment strategy in patients exhibiting visceral metastasis (21). However, upon the termination of chemotherapy for metastatic disease, disease progression occurs quickly. For example, studies conducted by Park *et al* (22) and Alba *et al* (4) demonstrated that the median PFS time following chemotherapy termination was 3.8 and 5.1 months, respectively. Therefore, it is important that maintenance therapy for MBC patients is conducted. If a patient exhibits a hormone receptor-positive tumor, the majority of healthcare workers would initiate treatment with maintenance hormonal therapy following the completion of chemotherapy, despite the lack of prospective randomized trials regarding its efficacy (23). However, for patients with HR-negative tumors, endocrine-resistant disease of the luminal subtype or rapidly proliferative and/or symptomatic disease, there is no preferred method for maintaining stable disease. Recently, the Korean Cancer Study Group conducted a phase III clinical trial of HER2-negative MBC patients who had achieved disease control following six cycles of first-line paclitaxel/gemcitabine chemotherapy (22). The study determined that subsequent gemcitabine/paclitaxel maintenance chemotherapy was associated with a statistically significant increase in the median and 6-month PFS rates, as well as an increase in the overall survival period (22). Furthermore, single-agent chemotherapy was considered to be an effective maintenance treatment and was the preferred choice compared with combination agents.

The present study considered DX chemotherapy to be the preferred treatment stratetgy for MBC patients due to its positive response and tolerable side effects. In 2002, O’Shaughnessy *et al* (14) conducted a phase III study comparing the effects of docetaxel administraton alone with docetaxel in combination with capecitabine (TX chemotherapy). The addition of capecitabine to docetaxe treatment resulted in an extended time to disease progression, improved overall survival and more manageable side effects. Similarly, a PFS time of 11 months for TX chemotherapy was determined in the present study. Additionally, the total and maintenance PFS times were similar between the capecitabine and HRT maintenence groups (10.9 and 6.8 months vs. 11.1 and 5.8 months). Approximately half of the patients maintained their response to combination chemotherapy for >6 months and achieved a clinical benefit in regardless of whether they were in the capecitabine or HRT maintenence group; however, six patients received HRT for maintenance treatment >12 months and two patients for >20 months, while four patients received capecitabine >1 year. The improved response in the HRT group may be because HRT is better tolerated compared with capecitabine. For the 49 patients who did not undergo palliative endocrine therapy, the use of HRT for maintenance therapy demonstrated a longer PFS time (11.5 months vs. 6.1 months), consistent with previous reports (6,24). Additionally, of the 30 patients who received HRT as first-line metastasis treatment prior to TX chemotherapy administration, the capecitabine maintenance group exhibited a higher PFS compared with the HRT maintenence group. This significant reduction in PFS (P=0.043) may be associated with endocrine resistance caused by repeated HRT (25–27). In the present study, ~70% patients were postmenopausal; and according to the results of several clinical trials, postmenopausal advanced breast cancer patients are initially recommended to undergo endocrine therapy predominantly consisting of a nonsteroidal (letrozole or anastrozole) or steroidal (exemestane) aromatase inhibitor (28–30). However, even if this type of hormonal therapy is initially effective, it considered to be ineffective following relapse caused by acquired resistance (26). According to the results of the present study, capecitabine may be an optional maintenance treatment for patients who are resistant to endocrine therapy.

Numerous trials have been conducted that indicate that the use of continuous chemotherapy for the treatment of breast cancer prolongs the duration of remission; however, its effect on quality of life and survival are less consistent (3,31,32). Recently, a meta-analysis was conducted, which analyzed the data from 11 randomized trials. A longer duration period of first-line chemotherapy was associated with a markedly improved PFS period (5); however, it is essential that the appropriate agent is selected for maintenance treatment by considering its impact on quality of life and the extent of toxicity, against the improvement in disease-associated symptoms and the benefits of tumor regression. Using these considerations, capecitabine was selected as an appropriate candidate agent for patients who responded to initial TX chemotherapy. The current study indicated that single-agent capecitabine maintenance treatment was well tolerated and its ability to be orally administered avoids the need for a central venous device, thus, reducing discomfort and the risk of developing a central venous catheter infection. Furthermore, the use of oral capecitabine reduces the hospitalization and administration costs and appears to improve the patient quality of life.

In conclusion, the results of the present study indicate that single-agent capecitabine maintenance therapy may be an a potential therapeutic strategy for MBC patients who have responded to capecitabine-based chemotherapy prior to disease progression. In particular, capecitabine may offer a more effective maintenance treatment duration compared with HRT for patients who have previously undergone first-line palliative HRT for MBC.

## Figures and Tables

**Table I:** Baseline characteristics of all patients (n=79).

	Capecitabine maintenance		HRT maintenance		
			
Characteristics	n	%	n	%	P-value
Menopause status					0.406
Pre	12	30.8	9	22.5	
Post	27	69.2	31	77.5	
ECOG PS					0.372
0	23	59.0	30	75.0	
1	14	35.9	9	22.5	
2	2	5.1	1	2.5	
HR status					0.082
Positive	28	71.8	35	87.5	
Negetive	11	28.2	5	12.5	
Lymph nodes, n^a^					0.516
0–3	28	71.8	26	65.0	
≥4	11	28.2	14	35.0	
Her-2 status					0.876
Positive^b^	6	15.4	5	12.5	
Negative^c^	32	82.1	34	85.0	
Unknown	1	2,4	1	2.5	
Metastatic site					
Liver	14	35.9	12	30.0	0.577
Lung	23	59.0	18	45.0	0.214
Bone	20	51.3	21	52.5	0.914
Brain	3	7.7	4	10	1.000
Soft tissue	22	56.4	30	75.0	0.082
Visceral metastasis					0.210
Yes	32	82.1	28	70.0	
No	7	17.9	12	30.0	
Metastatic sites, n					0.943
1	7	17.9	8	20.0	
2	18	46.2	17	42.5	
≥3	14	35.9	15	37.5	
Disease-free interval, years					0.539
<2	13	33.3	16	40.0	
≥2	26	66.7	24	60.0	
Prior adjuvant chemotherapy					0.523
Taxane	20	51.3	20	50.0	
Anthracycline	35	89.7	27	67.5	
Prior adjuvant endocrine therapy	25	64.1	27	67.5	0.764
Prior palliative endocrine therapy	17	43.5	13	32.5	0.310

a Lymph nodes, n indicates the number of metastatic lymph nodes;

b HR-positive status indicates estrogen and/or progesterone receptor-positive;

c HR-negative status indicates estrogen and progesterone receptor-negative.

HRT, hormone replacement therapy; ECOG PS, Eastern Cooperative Oncology Group Performance Status; HR, hormone receptor; Her-2, human epidermal growth factor receptor-2.

**Table II:** Treatment-associated toxicities.

Adverse event	Capecitabine maintenance, n (%)	HRT maintenance, n (%)	P-value
Neutropenia, grade			0.492
0	17 (43.6)	23 (57.5)	
1	4 (10.3)	3 (7.5)	
2	10 (25.6)	10 (25.0)	
3	8 (20.5)	4 (10.0)	
4	0 (0.0)	0 (0.0)	
Vomiting/diarrhea, grade			0.433
0	27 (69.2)	29 (72.5)	
1	6 (15.4)	7 (17.5)	
2	3 (7.7)	4 (10.0)	
3	3 (7.7)	0 (0.0)	
4	0 (0.0)	0 (0.0)	
Hand-foot syndrome, grade			0.052^a^
0	20 (51.3)	29 (72.5)	
1	7 (17.9)	3 (7.5)	
2	4 (10.3)	2 (5.0)	
3	8 (20.5)	6 (15.0)	0.521^b^

HRT, hormone replacement therapy.

a Mean incidence of hand-foot syndrome (48.7 vs. 27.5%).

b Mean incidence of grade III toxicity hand-foot syndrome.
